# Direct Observation (DO) for Drug-Resistant Tuberculosis: Do We Really DO?

**DOI:** 10.1371/journal.pone.0144936

**Published:** 2015-12-29

**Authors:** Stella Benbaba, Petros Isaakidis, Mrinalini Das, Sonakshi Jadhav, Tony Reid, Jennifer Furin

**Affiliations:** 1 Médecins Sans Frontières, Mumbai, India; 2 Médecins Sans Frontières, Operational Research Unit, Luxembourg City, Luxembourg; 3 Tuberculosis Research Unit, Case Western Reserve University, Cleveland, Ohio, United States of America; Fundació Institut d’Investigació en Ciències de la Salut Germans Trias i Pujol, Universitat Autònoma de Barcelona, SPAIN

## Abstract

**Introduction:**

Directly-observed therapy (DOT) is recommended for drug-resistant tuberculosis (DR-TB) patients during their entire treatment duration. However, there is limited published evidence on implementation of direct observation (DO) in the field. This study aims to detail whether DO was followed with DR-TB patients in a Médecins Sans Frontières (MSF) tuberculosis program in Mumbai, India.

**Methods:**

This was a cross-sectional, mixed-methods study. Existing qualitative data from a purposively-selected subset of 12 patients, 5 DOT-providers and 5 family members, were assessed in order to determine how DO was implemented. A questionnaire-based survey of DR-TB patients, their DOT-providers and MSF staff was completed between June and August 2014. Patients were defined as”following Strict DO” and “following DO” if a DOT-provider had seen the patient swallow his/her medications “every day” or “most of the days” respectively. If DO was not followed, reasons were also recorded. The qualitative data were analysed for theme and content and used to supplement the questionnaire-based data.

**Results:**

A total of 70 DR-TB patients, 65 DOT-providers and 21 MSF health staff were included. Fifty-five per cent of the patients were HIV-co-infected and 41% had multidrug-resistant-TB plus additional resistance to a fluoroquinolone. Among all patients, only 14% (10/70) and 20% (14/70) self-reported “following Strict DO” and “following DO” respectively. Among DOT-providers, 46% (30/65) reported that their patients “followed DO”. MSF health staff reported none of the patients “followed DO”. Reasons for not implementing DO included the unavailability of DOT-provider, time spent, stigma and treatment adverse events. The qualitative data also revealed that “Strict DO” was rarely followed and noted the same reasons for lack of implementation.

**Conclusion:**

This mixed-methods study has found that a majority of patients with DR-TB in Mumbai did not follow DO, and this was reported by patients and care-providers. These data likely reflect the reality of DO implementation in many high-burden settings, since this relatively small cohort was supported and closely monitored by a skilled team with access to multiple resources. The findings raise important concerns about the necessity of DO as a “pillar” of DR-TB treatment which need further validation in other settings. They also suggest that patient-centred adherence strategies might be better approaches for supporting patients on treatment.

## Introduction

The treatment for drug-resistant tuberculosis (DR-TB) is difficult: it is lengthy with a high pill burden, substantial toxicities, poor outcomes and challenging social factors including stigma and financial burden. These factors contribute to high lost-to-follow-up (LTFU) rates. To prevent LTFU, the current international recommendations for DR-TB treatment stress the importance of directly-observed therapy (DOT) for all patients. Direct observation (DO) means a designated provider is supposed to observe a patient swallowing his/her medications on a daily basis throughout the entire treatment course [1–4]. For DR-TB programmes, the WHO endorses guaranteed DO services to all patients at least six days a week, with an extended timetable to allow the delivery of treatment twice a day if prescribed [1]. All countries treating DR-TB report using DOT [5], but there are few data about how DOT implementation is actually performed in the field.

The DO strategy is resource-intensive for patients, providers, and programs and, in fact, there is limited evidence to support the role of DO in successful treatment of TB [6,7]. There may be alternative strategies to promoting adherence in DR-TB treatment: it is noteworthy that in the WHO’s ‘Draft Post-2015 Global Tuberculosis Strategy Framework’ there is no overt mention of DO, with the focus instead moving towards patient-centred care [8]. Some recent qualitative evidence also suggests that the static concept of “an adherent patient” also merits exploration, given that patients who are co-infected with HIV and DR-TB tend to preferentially adhere to their antiretroviral treatment while compromising their adherence to DR-TB treatment [9]. Unfortunately, the concept of DO is so ingrained in the paradigm of DR-TB therapy, there has been little frank discussion of its utility to date.

In India, the Revised National Tuberculosis Control Program (RNTCP) strongly promotes direct observation for the millions of individuals diagnosed with TB in the country, dedicating significant human and financial resources to this practice. Little is known, however, about how DO functions in the field and the role it may play in the successful treatment of DR-TB in the Indian setting. Médecins Sans Frontières (MSF) has been treating DR-TB in Mumbai since 2007, initially focusing on HIV co-infected patients. DO is used throughout the treatment and it is complemented by intensive patient education, individualised adherence counselling and psychosocial support to patients and their family caregivers. However, previously collected qualitative data from patients and providers and frequent anecdotal reports from outreach nurses indicated that some of the patients in the MSF cohort were not, in fact, receiving DO. Thus, the goal of this study was to assess and describe the implementation of DO within the context of the MSF Mumbai treatment program.

## Methods

### Study Design

This was a mixed-methods study involving a two phase sequential design. We first looked at previously collected qualitative data from a purposively-selected subset of patients [3,4]. The findings of the first phase of the study related to DO informed the design of the second phase of the study which included a quantitative and a small, complementary qualitative component. The quantitative component of the study deployed a cross-sectional questionnaire-based survey between July and August 2014. The complementary qualitative component involved collecting and analysing new qualitative data from key informants between July and August 2014.

### Study population

Existing data from 12 DR-TB patients co-infected with HIV, five care-providers and five family members who were purposively selected from a 2013 cohort were included in the study. The study population was previously described [3].

All DR-TB patients who were receiving treatment through the MSF clinic during the study period and their paired DOT-providers and MSF health staff who consented to participate were included in the quantitative cross-sectional survey between July and August 2014. We also included patients who had stopped treatment during the three months prior to the study.

Three key informants were purposively selected for their knowledge on DO and were interviewed in Mumbai, India during the same period (July—August 2014).

### Treatment and follow-up protocol

All patients receive an individualized regimen according to their DR-TB resistance profile. The first 8–12 months of the treatment include an injectable drug, intensive phase (IP), while the remaining 18–20 months include only oral medications, continuation phase (CP). Treatment is delivered through an ambulatory, community-based program. Patients are evaluated by a multi-disciplinary team of trained physicians, nurses, social workers and a psychologist at the MSF clinic. The same team follows the patient monthly throughout their treatment. An MSF Outreach Nurse works with the patient to identify a health worker at the community level to provide DO once a day at least six days per week. Where DO is provided at a clinic, this is available no more than 10 minutes walking distance from patients’ homes. When a nurse is the provider, they visit the patient daily at their home.

All DOT-providers are trained to provide DO, administer injections and monitor for adverse effects. MSF supplies the providers with second line TB drugs, materials to give injections, N95 respirators and training on infection control. Each DOT-provider is contacted regularly by phone and monthly by an MSF staff visit. Patients come monthly to the MSF clinic for medical and psychosocial follow-up. All treatment is provided to the patient free of charge.

### Data Collection and Analysis

#### Qualitative data

The qualitative data were derived from two sources. The first source was from an in-depth interview set collected previously between May-September 2012 as previously described [3,4]. This set of interview data was used to identify relevant themes of DO and to inform the design of the questionnaires for the cross-sectional quantitative study and the content of the interview guides for the key informant interviews.

During the second phase, complementary qualitative study, we interviewed key informants with specialised knowledge and expertise on the realities of DO implementation and applicability for DR-TB treatment in the Mumbai context. We chose informants who would incorporate views from both the private and public health sectors. We interviewed a prominent private chest physician, a District TB Officer representing RNTCP and a high-ranked officer from the Care, Support and Treatment department of MSACS (Maharashtra State AIDS Control Society). Interviews were recorded using a digital recorder and transcribed. Notes were taken in one case where the key informant did not wish to be recorded and were expanded immediately afterwards.

#### Quantitative data

We used an identical definition for DO (“a patient being directly observed by a provider swallowing his/her medications on a daily basis throughout the entire treatment course”) for patients, providers and MSF staff. Patients were defined as “following Strict DO” and “following DO” if a DOT-provider had seen the patient swallow his/her medications “every day”‘ or “most of the days” (at least 4 out of the last 7 days of the previous week) respectively. If DO was not followed, reasons were recorded through the provision of a check-list of responses and an option for ‘other’ if there was a novel reason expressed.

The study questionnaires were piloted and after finalization they were administered to three distinct groups: patients, DOT-providers and MSF health staff. All patient and MSF health staff questionnaires were administered at the MSF clinic in Mumbai. DOT-provider questionnaires were completed at each individual location of the provider. The questionnaires employed were paired (patient/DOT-provider), quantitative and descriptive. Patient and DOT-provider questionnaires were facilitated by one and two interviewers respectively, all fluent in Marathi, Hindi and English to maintain consistency: the interviewers were not directly involved in the care of the patients or previously linked to the DOT-providers. The MSF health staff questionnaires were facilitated in a group meeting and their responses were anonymous. The questionnaires were identical with the ones used for the pairs of patients/DOT-providers but the MSF staff was asked to report on the overall cohort and not on the individual patients and providers.

Both the questionnaires and the interviewer emphasized the oral medication so as to reduce automatic answers related only to seeing a health professional for receiving the daily injection in the intensive phase. To reduce the risk of social desirability bias the specific terminology of ‘DOT’ or ‘DO’ was not used. Patients were asked to mark their overall adherence on a simple scale for the IP and CP phases of the treatment. They also nominated a category for the way they took their medicine in the previous week overall, and then they were asked to recall specifically day by day in the previous week how they took their medicines. DOT-providers were asked exactly the same questions about the same time period but with the day-by-day recall omitted as we found during the pilot study that this was not practical. A cover sheet was also completed by the interviewer with information taken from the patient’s file related to demographics, treatment, HIV status and DOT-provider details. Finally we used the individual patient medical records kept at the MSF clinic to obtain demographic and clinical data as well as interim and final treatment outcomes.

#### Data analysis

A thematic analysis was performed to analyse the qualitative data in the study. The data transcripts were read and reviewed manually by one of the investigators (JF). A second investigator (PI) reviewed the transcripts to enable cross-validation of the themes identified.

Differences in interpretation were discussed and resolved by the two investigators.

Quantitative data were analysed using descriptive statistics. The chi-square test was used to examine differences in characteristics between patients who followed DO and those who did not. Poisson regression models were used to explore associations between demographic, clinical and programmatic characteristics of the patients and adherence to DO.

### Ethics

This study was approved by the Médecins Sans Frontières independent Ethics Review Board, Geneva, Switzerland. The qualitative aspect was approved by the Institutional Review Board of the Maharashtra Association of Anthropological Sciences—Centre for Health Research and Development (MAAS-CHRD), Pune, India. All participants provided written informed consent prior to involvement. We obtained written informed consent from the next of kin, caretakers, or guardians on behalf of the children enrolled in the study.

## Quantitative & Qualitative Findings

### Patient Characteristics

A total of 70 DR-TB patients, 65 DOT-providers and 21 MSF health staff were included in the questionnaire-based survey. Fourteen per cent of the patients were children (≤ 15 years) with the mean age of the cohort at 30 years (range: 11–76) and 46% were male. Fifty-five per cent of the patients were co-infected with HIV and 41% had DR-TB that was also resistant to a fluoroquinolone or an aminoglycoside. Most of the patients (78%) were registered for DO at a nearby clinic; the others were receiving DO at home with a nurse (10%) or from the hospice/orphanage in which they were residing (11%). Characteristics of these patients are summarised in Table 1 while the characteristics of the subset of patients of the qualitative study were described elsewhere [3].

**Table 1:** Socio-demographic and clinical characteristics of patients receiving DR-TB treatment in Mumbai, India, 2014.

Characteristics	DR-TB patients (N = 70)
	n (%)
**Age** (years)	
0–15	10 (14.3)
16–25	17 (24.3)
26–35	20 (28.6)
36 and above	23 (32.9)
**Sex**	
Male	32 (45.7)
Female	38 (54.3)
**Education**	
Illiterate	3 (4.3)
Primary	25 (35.7)
Secondary	35 (50.0)
Graduate	7 (10.0)
**HIV co-infected**	38 (54.3)
**DR-TB resistance pattern**	
MDR	17 (24.3)
Pre-XDR	29 (41.4)
XDR	19 (27.1)
X-XDR	5 (7.1)
**Treatment phase**	
Intensive phase	33 (47.1)
Continuation phase	33 (47.1)
Finished treatment	4 (5.7)
**DOT-provider**	
Clinic (Clinician/Non-clinician)	55 (78.6)
Nurse (Home-visit)	8 (11.4)
Hospice/Orphanage	7 (10.0)

### Strict DO, DO and non-DO

Among all patients and across full treatment duration, only 14% (10/70) and 20% (14/70) self-reported “following Strict DO” and “following DO” respectively. Among DOT-providers, 46% (30/65) reported that their patients followed any DO, either strict or not. MSF health staff reported that none of the patients in fact followed any DO.

**Fig 1:**
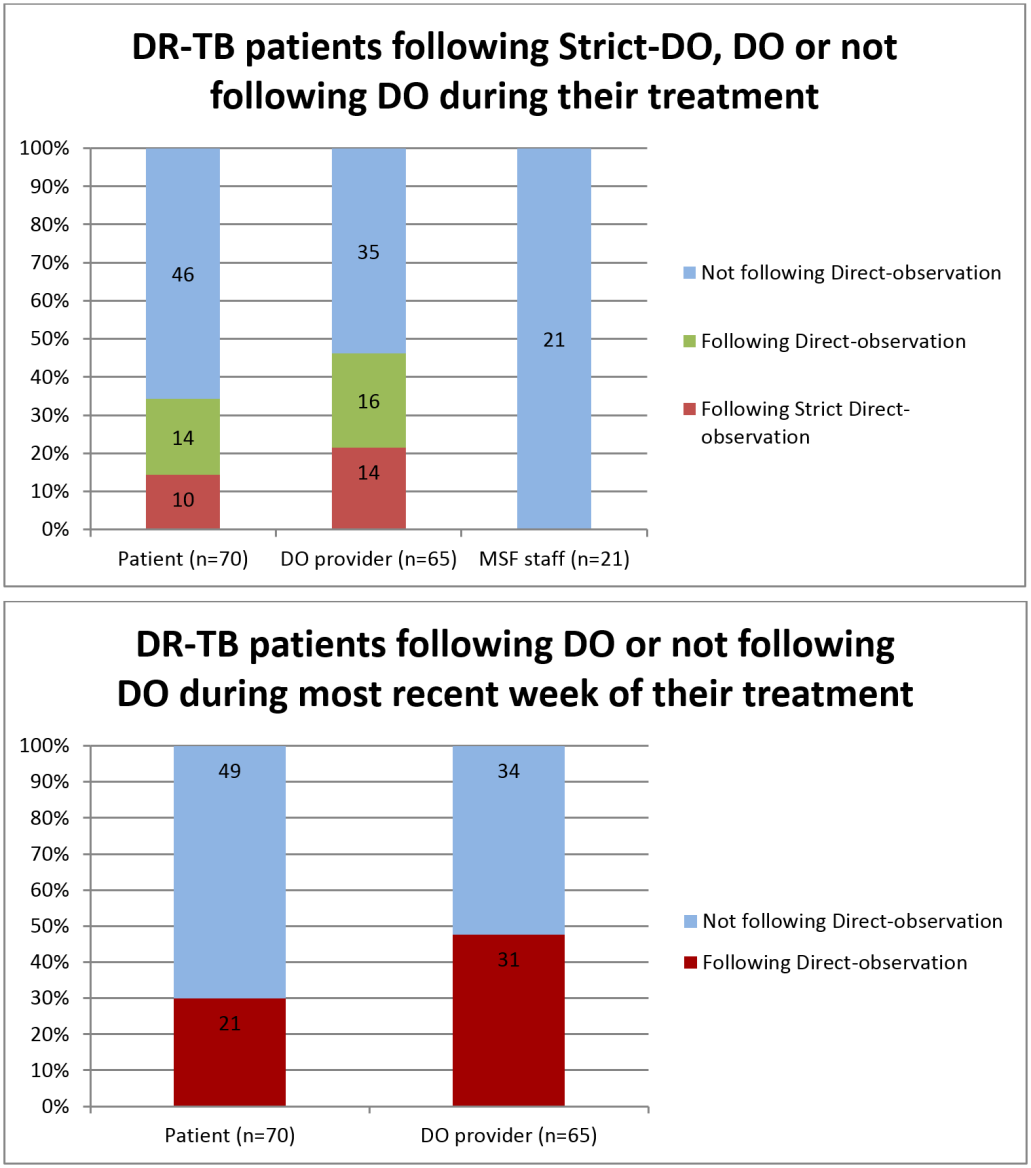
Direct Observation (DO) or non-DO as reported by patients, DOT-providers and MSF staff. (A) DR-TB patients following Strict-DO, DO or not following DO during their treatment. (B) DR-TB patients following DO or not following DO during most recent week of their treatment.

There was no significant difference between the patient reports of receiving DO during IP or CP (42% and 45% respectively) whereas the DOT-providers reported 60% delivery of DO in IP and only 47% in CP. The perception of the MSF health staff was that 38% of patients received DO in IP phase but that none received DO in CP. Fig 1A and 1B illustrates the proportions of patients who reported adhering to DO along with the perceptions of DOT-providers and MSF health staff.

There were no demographic or clinical factors significantly associated with non-adherence to DO as shown in Table 2. There was no difference in treatment outcomes (interim and end of treatment outcome) between patients following and not following DO as shown in Table 3a and 3b.

**Table 2:** Demographic and clinical factors associated with DR-TB patients not receiving ‘directly observed’ treatment in Mumbai, India, 2014.

Explanatory Variable	Patients without directly observed treatment^┼^ (N = 46), n (%)	Patients with directly observed treatment^┼^ (N = 24), n (%)	Chi-square/t-test (p-value)	aPR^a^ (95% CI)
**Age** (years, median, IQR)	33 (21–42)	25 (17–35)	0.09	1.00 (0.99–1.01)
**Sex**				
Male	21 (65.6)	11 (34.4)	0.99	0.96 (0.83–1.09)
Female	25 (65.8)	13 (34.2)		
**HIV**				
Infected	25 (65.8)	13 (34.2)	0.99	0.99 (0.87–1.14)
Non-infected	21 (65.6)	11 (34.4)		
**DR-TB resistance pattern**				
MDR	10 (58.8)	7 (41.2)	-	-
Pre-XDR	19 (65.5)	10 (34.5)		
XDR	14 (73.7)	5 (26.3)		
X-XDR	3 (60.0)	2 (40.0)		
**DOT-provider**				
Clinic (Clinician/Non-clinician)	37 (67.3)	18 (32.7)	0.59	1.03 (0.88–1.20)
Other than Clinic	9 (60.0)	6 (40.0)		

^┼^Row percentage in parenthesis; IQR: Inter-quartile range;

^a^aPR: adjusted Prevalence Ratios (calculated by Poisson regression).

**Table 3:** Association between Direct Observation (DO) and interim and end of DR-TB treatment outcomes, Mumbai, India 2014

**A. Interim outcomes (culture conversion to negative)**			
	**Favourable outcome** * **(N = 63), n (%)**	**Unfavourable outcome** ** **(N = 7), n (%)**	**Fisher-exact test (p-value)**
**DO**	21 (88)	3 (12)	0.45
**Non-DO**	42 (91)	4 (9)	
**B. End-of-treatment outcomes**			
	**Favourable outcome** ^┼^ **(N = 24), n (%)**	**Unfavourable outcome** ^┼┼^ **(N = 6), n (%)**	**Fisher-exact test (p-value)**
**DO**	10 (83)	2 (17)	0.54
**Non-DO**	14 (78)	4 (22)	

DO: Following direct observation either every day or more than 4/7 week-days during treatment; Non-DO: Not following direct observation;

*Favourable outcome: Culture conversion to negative at the end of intensive phase;

**Unfavourable outcome: Culture not converted;

^┼^Favourable outcome: Patient cured or completed treatment;

^┼┼^Unfavourable outcome: Patient died or was lost-to-follow-up during treatment

### Reasons for non-DO

Multiple reasons were given by patients, providers, and MSF staff for not following DO. Table 4 provides a detailed, quantitative account of these responses ranked by frequency and stratified by responder.

**Table 4:** Common reasons for Non-direct Observation (non-DO) for DR-TB patients on treatment in Mumbai, India, 2014.

Common reasons*	Patient responses (N = 70), n (%)	DOT-provider responses (N = 65), n (%)	MSF health staff responses (N = 21), n (%)
1	DOT-provider not available (28, 40%)	DOT-provider trusts the patient to take medicine without being directly-observed. (19, 29%)	DOT-provider trusts the patient to take medicine without being directly-observed. (14, 68%)
2	Adverse events (18, 26%)	Time consuming for patient (14, 22%)	Heavy workload of DOT-provider (13, 62%)
3	Time consuming for patient (12, 17%)	Adverse events (10, 15%)	Patient’s job (13, 62%)
4	Opinion of other people in their community (7, 10%)	DOT-provider is unavailable (9, 14%)	Time consuming for patient (12, 57%)
5	Opinion of other patients in same clinic (7, 10%)	Heavy workload of DOT-provider (8, 12%)	Adverse events (12, 57%)

* Study participants were able to provide more than one reason.

The thematic analysis of the qualitative data revealed “negative” and “positive” reasons why DO could not be followed. The negative reasons fell into 3 main categories: 1) lack of accessibility for patients, including time constraints and clinic hours; 2) promotion of stigma through DO; and 3) concerns about managing adverse events during the administration of DO. “Positive” reasons were reasons why it was felt that DO was not necessary and therefore did not need to be done. Positive reasons included: 1) flexibility and understanding among caregivers and “tolerance” among programme-managers, 2) a sense of trust between providers and patients, and 3) the existence of family or social support.

#### Negative reasons

*1) Lack of accessibility*: Lack of accessibility was a main reason given by participants for not following DO. A commonly reported problem was the absence of providers when patients went to receive DO.

*Patient*: *“Yes … it has happened that the doctor (DOT-provider) did not come so I missed the injection as well as the pills*.*”*

*Interviewer*: *“So that was because the doctor did not come*. *Has it happened due to a fault of yours*?*”*

*Patient*: *“No I have never missed a dose on my account*. *I told the counsellor*. *He said it should not happen again*. *If the doctor (DOT-provider) has to be away the next day then the previous day he gives me the injection and pills*. *He had not done it then so I missed the dose*.*”*—Patient 6

Several patients also reported missing doses of medications because they had to attend appointments with other physicians, including doctors for HIV and diabetes treatment. DO for TB and DR-TB was totally vertical and in isolation from any other health-service, even in clinics where HIV and TB services were totally integrated, such as the MSF clinic.

*“When I go there (MSF clinic for my HIV appointment)*, *it is hard to take first the injection and pills and then go there*. *I feel dizzy and nauseous; I vomit sometimes and I have to travel far to get here*. *So on that day I miss the dose*.*”—Patient 13*

Patients often lamented the possibility of resuming a normal life and work, especially given the requirement for DO; the administration of drugs became a daily burden and the daily life and work routine had to be built around the time of DO.

*“I left home in this morning*, *travelled to come here*, *will take medicines at [the DOT facility] and then go to work directly*. *Then I will return home at midnight*. *See how difficult it will be for a man to take the medicines and work*.*”—Patient 9*

The absence of a provider was a common enough occurrence that many patients made “contingency plans” for taking treatment when their DOT-providers were not around. Such contingency plans are common among HIV patients on antiretroviral treatment but they are not part of any national TB or DR-TB treatment programme strategies.

*“An extra packet is kept here at home which the doctor has given for the times when he is not there so that he can at least take the pills*. *The injection is not given but a pill packet is given*.*”—Family care provider to Patient 1*

Of note, patients reported travelling all the way to the clinic to receive DO but instead got self-administered therapy while sitting in the clinic. Reporting to a clinic or other facility was seen by DOT-providers as a substitute for DO. One should question the “honesty” of such a strategy that requires patients to go to a facility for a “daily drug prescription”.

*“No the doctor is around there*. *He has to see other patients so he gives the medicine and I have to sit outside and eat it*. *He also has a business to run and cannot sit around so long for me*. *He says eat the pills and that’s all*.*”—Patient 2*

*2) Promotion of stigma through DO*: Patients, providers, and family members all reported that DO was difficult because they felt it reinforced or perpetuated stigma. This was particularly felt among HIV/DR-TB co-infected patients who had experienced stigma earlier as HIV patients at the HIV clinics, and then again while receiving treatment for DR-TB. The interviews often revealed embarrassment and loss of self-respect among patients taking DO at a doctor’s clinic:

*“The way to take medicines is that I go to the doctor*, *if there are 4–5 customers over there*, *and I would take medicines in front of them then they will question me what is this medicine for*? *The doctor will tell them that this man has HIV*, *then the way other persons look at you changes*, *people try to keep away from you and sometimes I would vomit*, *or bring out sputum then they would pinch their noses*, *so I thought it is better that I took medicines at some distant place”—Patient 8*

The perception that DO enforces stigma was also reported by family members or other caregivers who were dismayed by the reactions of neighbours, relatives, and other patients at the waiting areas of private clinics which served as DO facilities.

*“Yes*. *He does not eat them there because many of our neighbours are around in the clinic and if he vomits or is dizzy after taking the pill it will be embarrassing for him*.*”—Family care provider to Patient 1*

*3) Concerns about managing adverse events*: Several patients and providers reported that DO was not done because it made things difficult for the patient if there were adverse events. Adverse events, especially nausea, vomiting and dizziness which were extremely common, especially during the first period of the treatment, made the DR-TB patients “visible” to other patients at the DO facilities and induced stigma as earlier described. But adverse events, especially the ones that occur directly after the swallowing of the pills were moreover disabling and incapacitating. In several circumstances the DOT-providers and patients “agreed” to skip DO as it was not easy for the patient.

*“My house is on a hill and the dispensary at the bottom and it takes me 10 minutes to get there*. *It tires me to go there so I get the injection at the doctor’s and then take the pills home and to take them later otherwise I get dizzy and it gets late to return home*. *I take the medicines after I reach home*. *The doctor has helped me a lot by doing this*. *Otherwise they say the pills have to be taken at the doctors place itself*. *They say that*. *There are patients in the clinic*, *every day if you come there and take the pills and vomit*, *it does not look nice so he gave me the pills to take at home*.*”—Patient 6*

This was also reported by providers as a reason for not giving DO.

*“But he takes the medicines home—he had told me that he would like to take these at home because he might vomit etc*. *He informs me immediately if he vomits after taking medicines*. *But I see the result of the medicines*.*”—DOT-provider 2*

#### Positive Reasons

*1) Flexibility and understanding among DOT-providers and programme-managers*: The key informants who were interviewed for the study were aware of “alternatives to DO” practices in the field and in fact were very open and understanding. They often used the terms “flexibility” and “support” to describe what they thought was needed to ensure adherence to treatment. The need for counselling was a theme that was recurrently emerging during the interviews.

*“Even I observed*, *counselling is the only way*. *It is the only tool to decrease everything*. *It is the tool to make the people adhere to treatment*, *the tool to reduce defaulters*, *the tool to do infection control*. *Otherwise it is not possible only*.*”—Key Informant 2*

*“It (DO) is not necessary if the patient is counselled properly*. *Maybe it is relevant in the beginning*, *until they understand their medication and are used to the routine*, *and then no need*.*”—Key Informant 1*

*“…there should be some kind of flexibility*, *because people are not one size fits all*. *There are different levels of commitment in adherence in different people*. *That’s why counselling as such*, *when we have an experience in HIV/TB also*, *here DOTS is just an extended therapy*. *But in HIV you have to have a life-long therapy*. *When we look at these things*, *one*, *it is not possible to give a life-long therapy as directly observed*, *secondly even if you do try to give directly observed in such extended situations in life*, *it is very difficult to maintain*.*”—Key Informant 3*

The extended period required for DR-TB treatment makes daily DO unrealistic. Life events were often described as examples of such unrealistic demand being put on patients, providers and systems. In fact the strictness of DO and the lack of supportive counselling were considered important reasons for the high lost-to-follow-up (“default”) rates observed among DR-TB patients. Thus a need for a flexible approach was often expressed as being essential for adherence to treatment.

*“Another reason for defaulter in Mumbai—there is a sudden death in a family member*, *they don’t have time to come {…} to tell they are going to leave to their native place*. *This type of people default {*…*} we can tell to send their relatives to take 7 days medication which can be carried to the native place*. *So that we can at least help that person to decrease the number of days of defaulting*.*{…} The system says don’t give medication*. *System is telling*. *But what about defaulting*? *If you give medication with them*, *patient won’t default*. *So*, *there has to be flexibility in the programme*, *otherwise we will not be able to control this default rate because of uncontrolled circumstances which happens in life*. *Sudden death*, *marriage ceremony*, *festivals*.*”—Key Informant 2*

*2) Patient “trusted” to perform self-administered therapy*: A patient-centred approach was spontaneously adopted by some DOT-providers and this practice was supported by patients, family members and programme managers. The “one-size-fits-all” strategy was questioned and as Key Informant 3 said:

*“If there is specialized attention given for these people I think it would definitely give a better outcome*.*”—Key Informant 3*

Patients were sometimes trusted to be given the medicines and DO was not considered relevant any more. Adherence to treatment became more of an agreement, a contract between the patient and the caregiver.

*“It will depend on his rapport with the particular DOT provider*. *If the DOT provider feels that this patient is very sincere*, *honest*, *and he will take the medication*, *then he may give him for 7 days*, *or whatever it is*, *or 2 days or 3 days*. *{…}That can be arranged*, *it is a local arrangement which is done*.*”—Key Informant 2*

Private practitioners in Mumbai do not require DO; instead the focus is on counselling and patient education. This might be one of the reasons for the “appeal” of the private sector to patients.

*“When starting treatment for a patient with DR-TB*, *we give them the full background of the importance of taking treatment*, *adherence and side effects*. *We spend extra time with them at the start of treatment to ensure they understand this*.*”—Key Informant 1*

*3) Family member or “treatment-buddy” provided support*: One additional reason why DO was not done by providers was that family members were entrusted with the job of ensuring that a patient took his or her medications. This was reported by several family members. An enabling family environment or the support by a ‘treatment-buddy’ were considered as more sustainable and convenient practice for treatment adherence, despite a reluctance by TB programmes to accept “family DO” as alternative to “facility DO” or “community DO”.

*“I make her sit in front of me and give her the medicines*. *I take her with me to see the doctor and get her to take her day time dose and the night time dose and the injection and then I go to work*. *By the time I come home it is 11pm*.*”—Family care provider to Patient 5*

As one key informant emphasized, the patients who are treated by private practitioners are in fact supported by family members or friends. Another key informant who represented the private sector went as far as to compare treatment outcomes between the two sectors and questioned the efficacy of DO and its association with better outcomes.

*“If the dose could be given to the family to observe at home—almost 40% of patients are from private sector*, *60% from public sector*. *40% patients—how they are supervised*? *Doctor doesn’t supervise*, *their family members are supervising*. *They are taking medication*, *it is not that they are defaulting*. *Why this cannot be followed in the public sector*?*”—Key Informant 2*

*“With counselling the DOT is not necessary*. *If you look at statistics from the national program and from the private sector for DR-TB the outcomes are about the same*. *25% defaulters*, *17% death*, *60% completed/cured*. *The default rate is comparable*, *but the reasons are different*. *In the private sector it is because they are shopping for other services due to lack of finance or migrating*. *In the public sector it is due to dissatisfaction with the service*, *not being treated kindly*, *inconvenience*.*”—Key Informant 1*

## Discussion

The results from this mixed methods study show that DO is not followed by a majority of patients during treatment for DR-TB in a cohort treated by MSF in Mumbai, India. This finding is noted among all respondents participating in the study, including patients themselves, family members, DOT-providers, and MSF health staff. Reasons for not doing DO were varied, but tended to fall into three negative categories—inaccessibility of services, perpetuation of stigma, and fear of adverse events—as well as three positive categories—flexibility and understanding, presence of trust and reinforcement of family and community members. This was a closely supported cohort, and the fact that a majority of patients did not report following DO suggests that the central tenet of DO as part of DR-TB treatment may be more of a reported phenomenon than an actual occurrence.

The model of therapy administration reported here shows that the field reality encompasses a more flexible approach. This flexibility allows for patients to access their medicines in a way that is more comfortable, convenient, and supported by treating staff and family members. This more accurately reflects what might be referred to as a “patient-centred” approach to DR-TB treatment, a concept that is receiving more attention in the medical and public health communities. What constitutes a patient-centred approach to care? Toczek et al. found that provision of treatment closer to the community level in a patient-centred manner contributes to improved retention in DR-TB care [10] and that there is an association between larger program size and increased risk of loss to follow-up.

In the setting of drug susceptible tuberculosis (DS-TB) it has been acknowledged that DO can be limiting for patients, is labour intensive to implement and moreover has not been proven to improve outcomes when compared to self-administered treatment (SAT) in controlled trials [11]. Also for DS-TB, a meta-analysis on prospective studies found that there was no difference between DO and SAT for microbial failure, relapse, or acquired drug resistance [12]. Despite the abysmal lack of evidence, DO is still being required in the context of second-line treatment due to factors already discussed related to toxicity, pill burden, length of treatment and also the risk to patient and community when doses are missed, increasing the chance of resistance amplification. As Pasipanodya and Gumbo suggest, the DOTS (Directly Observed Treatment Short-course) strategy is associated with unquestionable success [12]. Wright et al showed that community-based DO has a higher treatment success compared to clinic DO [13]. These findings however may be due to the infusion of resources, expertise, reliable provision of drugs and higher level of support to the patient rather than the direct supervision of watching a patient swallow their pills.

For patients co-infected with HIV and DR-TB, a study in South Africa found that patients perceived ART to be their own responsibility, whereas their DR-TB medications as the responsibility of the nurse. This lack of TB patient empowerment, perpetuated by treatment supervision (DOT), may contribute to the weakness of TB care [7].

A patient-centred approach to care is likely to be more effective in the treatment of patients with DR-TB than continuing to promote a façade of DO, which in essence requires mandatory daily reporting to a health facility in order to access care. However, the data in this paper could be interpreted as signalling the need for better reinforcement of DO as opposed to supporting the idea of a more flexible approach to adherence. We feel such a conclusion would be erroneous, however, given that this MSF program was highly resourced and had access to a number of strategies that should have ensured that excellent DO was achieved. The fact that even in this setting, DO could not be carried out suggests that it is not feasible under program conditions and that limited resources would be best directed elsewhere. Furthermore, the DO that was reported here seemed to place a heavy burden on patients and their families, who were often let down by the health system in the form of absent providers, increased stigma, and little attention given to the occurrence of adverse events.

There are several limitations to this study. First, both components of the study relied on relatively small samples of patients, family members, and caregivers, and therefore may not be generalizable to larger populations in different contexts. Second, the data collected were based on self-report of DO as opposed to actual observation of DO. In most studies of similar behaviour, however, participants tend to “over-report” the behaviour they feel is accepted as opposed to underreporting it. Thus, this study likely overestimates how often DO was actually done. Finally, many of the patients in this study also had HIV co-infection and may have been more familiar with self-administered therapy, thus biasing the results. In spite of these limitations, we feel the data presented here have important public health implications and point to the need for more sophisticated studies and analyses of DO and adherence in DR-TB.

## Conclusion

DO was not followed by two-thirds of the patients in this Mumbai DR-TB cohort. This relatively small cohort was supported and closely monitored by a trained, resource-heavy team and, moreover, counselling and adherence support were systematically offered to the patients. We therefore suggest that DO is likely followed even less often by patients in many programmatic settings with limited resources and with no adherence support. We believe it is imperative to explore alternatives to DO that are more patient centered and could potentially make better use of human and financial resources that are currently invested in a DO-only approach.

## Supporting Information

S1 Dataset Direct Observation for DR-TB Mumbai (XLSX)
